# Canopy seed survival through extreme fire in non‐serotinous conifers: An unexpected source of forest resilience

**DOI:** 10.1002/eap.70142

**Published:** 2025-11-17

**Authors:** Derek J. N. Young, Nina E. Venuti, David F. Greene, Andrew M. Latimer

**Affiliations:** ^1^ Department of Plant Sciences University of California, Davis Davis California USA; ^2^ Cal Poly Humboldt Arcata California USA

**Keywords:** disturbance, forest, germination, seeds, serotiny, wildfire

## Abstract

Across much of the semiarid conifer forests of western North America (“dry conifer forests”), the dominant tree species are non‐serotinous, lack soil seedbanks, and rarely disperse seeds much farther than 100 m, so tree regeneration in large, high‐severity burned patches is expected to be highly seed‐limited. Conifer seedlings do, however, sometimes establish at high densities deep within high‐severity patches in these forests, implying that seeds can sometimes survive intense wildfire even when all overstory trees die. Does seed survival in the canopies of non‐serotinous trees provide an unexpected source of forest resilience? To answer this question, we surveyed tree survival, fire severity, and seedling abundance across two very large wildfires in the first year after fire. Several of the study species had a good seed cone production year at the time of the fires. We stratified many of our plots deep within high‐severity patches far from surviving trees, where existing models predict regeneration failure due to lack of viable seeds. Contrary to such expectations, we found that conifer seedling densities in these areas were generally far greater than needed to replace the fire‐killed stand and sometimes approached seedling densities observed near surviving trees. Seedling densities in high‐severity areas far from surviving trees correlated negatively with local burn intensity (canopy foliage consumption), supporting the idea that the seeds originated locally and highlighting a critical driver of post‐fire recovery that is easily missed by traditional surveys conducted >2 years following fire. Seedling density was also strongly associated with burn date, suggesting that persistence of viable canopy seeds depends on synchrony between wildfire and cone ripening dates. Together, our results demonstrate that under the right conditions, canopy seed survival can lead to dense seedling establishment across large severely burned areas and may substantially support the resilience of dry conifer forests to the uncharacteristically severe fires that are becoming increasingly prevalent in this system.

## INTRODUCTION

In forests around the globe, wildfires are increasingly burning large contiguous patches at high severity, leaving vast areas without surviving reproductive trees (Coop et al., 2020; Nolan et al., 2022; Steel et al., 2018; Tran et al., 2020). In ecosystems that historically experienced substantial high‐severity fire, many plants have adapted traits that enable persistence through fire, including resprouting, soil‐stored viable seeds (“soil seedbanks”), and serotiny—the multiyear accumulation and aerial storage of cones containing ripe seeds whose release is triggered by fire (Keeley & Pausas, 2022). In contrast, dry conifer forests of western North America have historically experienced low‐ to moderate‐severity fire regimes and are dominated by tree species that lack serotinous aerial or soil seedbanks and cannot resprout. For these forests to recover from severe fire, therefore, current models assume that seeds must disperse from surviving trees into burned areas (Coop et al., 2020; Keeley & Pausas, 2022; Stephens et al., 2013; Stevens‐Rumann & Morgan, 2019). In accordance with this assumption, post‐fire regeneration of non‐serotinous conifers typically declines sharply as one moves from the edge of a high‐severity patch toward the interior (Davis et al., 2023; Greene & Johnson, 1996; Haire & McGarigal, 2010; Stevens‐Rumann & Morgan, 2019; and constituent work). Thus, the recent increase in large high‐severity burn patches has raised concerns that dry conifer forests will shift to persistent shrub‐ or grass‐dominated states (Coop et al., 2020; Millar & Stephenson, 2015).

**FIGURE 1:**
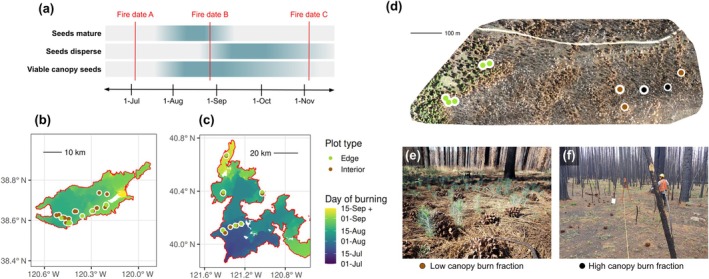
Canopy seed survival conceptual model and key environmental gradients evaluated. (a) Hypothetical timeline of reproductive phenology and its relation to the presence of viable canopy seeds. (b, c) Location of study plots within the Caldor Fire (b) and Dixie Fire (c), with background shading indicating burn date. (d) Drone‐derived orthomosaic from 2022 of a section of the Caldor Fire, exhibiting gradients between surviving green trees (green), killed trees with low canopy burn fraction (brown) and killed trees with high canopy burn fraction (gray‐black), and indicating the locations of five edge plots (green points), interior plots with low canopy burn fraction (brown points) and interior plots with high canopy burn fraction (black points). Plots with thick white rings are actual field plots included in the analysis; plots with thin white rings are illustrative only. (e) An example of a stand with low canopy burn fraction, with abundant yellow pine cones and seedlings. (f) An example of a plot with high canopy burn fraction, with yellow pine cones but few seedlings. The orthomosaic in panel (d) was produced from drone imagery collected by Samuel Ericksen. The photo in panel (e) was taken by Derek J. N. Young. The photo in panel (f) was taken by Alexis Ballman.

Some studies reveal, however, that conifer regeneration sometimes does occur in large high‐severity patches in dry conifer forests. Although conifer seedling densities far from surviving trees are often very low, they are sometimes high (e.g., >1000 seedlings ha^−1^) (Larson & Franklin, 2005; Pounden et al., 2014; Appendix S1: Figure S1) and poorly explained by live tree proximity (Gray & Franklin, 1997; Harris et al., 2020; Stewart et al., 2021). Researchers have speculated that a biological mechanism may produce these patterns: if seeds have matured but cone scales have not yet flexed open to release seeds, viable seeds may persist in the canopy and disperse after fire, even if the maternal parent is killed (Gray & Franklin, 1997; Harris et al., 2020; Larson & Franklin, 2005; Pounden et al., 2014; Figure 1a). Seed cones can be highly insulating (Fraver, 1992), even in non‐serotinous species (Greene et al., 2024; Lopez et al., 2025; Mercer et al., 1994; Michaletz et al., 2013). However, the implications of canopy seed survival in fire‐killed non‐serotinous conifers have only been explored theoretically (e.g., Lopez et al., 2025; Michaletz et al., 2013) and in a few field surveys, generally anecdotally. For example, several post‐fire forest regeneration studies that have reported high post‐fire seedling densities of Douglas‐fir (Gray & Franklin, 1997; Larson & Franklin, 2005), non‐serotinous lodgepole pine (Harris et al., 2020), and Engelmann spruce (Pounden et al., 2014) far from surviving trees proposed canopy seed survival as the likely mechanism.

Existing observations of possible canopy seed survival are essentially incidental, however, with the exception of one study that presents corroborating evidence that seeds originated from local, fire‐killed trees: a positive correlation between local cone density and local seedling density within a stand (Pounden et al., 2014). Thus, it remains unknown how widespread and ecologically important this forest regeneration mechanism is. With area burned at high severity increasing substantially in dry conifer forests (Abatzoglou et al., 2021; Williams et al., 2023), canopy seed survival in non‐serotinous conifers could have far‐reaching ecosystem implications, potentially enabling large areas of forest to regenerate more successfully than current ecological models predict.

In this study, we performed field surveys tailored to detect forest regeneration resulting from canopy seed survival in fire‐killed trees, particularly in the areas far from surviving trees typically expected to exhibit regeneration failure. Our two study fires, both among the 20 largest in US history, burned more than 4700 km^2^ over multiple months. We distributed survey plots in many distinct stands across much of the fires' extents (157 plots across 30 distinct areas), resulting in a study domain that extends from the southern Cascades to the central Sierra Nevada and represents a diversity of burn days (16 distinct burn days across a 50‐day span) and fire effects. We asked whether wildfire timing, wildfire intensity, and cone crop size explain forest regeneration via canopy seed survival and whether seed survival patterns vary by species. Within areas experiencing high‐severity fire that killed all reproductive conifers, we expected to find the highest seedling densities in areas where (1) fire burned in late summer when we expected ripe seeds to be present in tree canopies (Lopez et al., 2025); (2) the fire was not so intense as to fully consume conifer foliage (and thus presumably heat‐kill most viable seeds); and (3) the local cone crop in the year of fire (quantified as the density of cones found on the ground the following year) was greatest.

## MATERIALS AND METHODS

### Methods summary

To evaluate whether canopy seed survival may support dry conifer forest resilience to high‐severity fire, we extensively surveyed tree survival, fire severity, and seedling abundance in two anomalously large 2021 California wildfires, the Caldor and Dixie fires. We surveyed plots deep within high‐severity patches beyond the expected seed dispersal range of live trees (“interior plots”), as well as areas closer to the surviving forest edge (“edge plots”) to enable comparison of seedling densities near to and far from live adult trees. To evaluate the importance of the alignment of wildfire timing with tree reproductive phenology, we compared areas burned prior to expected seed maturity (“early‐burned”) to areas burned later in the season when we expected ripe seeds to be present in the canopy (“later‐burned”) (Figure 1a–c). To evaluate whether local variation in fire severity affects canopy seed survival, we also compared extremely high‐severity burned areas where foliage was incinerated (“high canopy burn fraction”) with high‐severity burned areas where foliage was killed and brown but still present (“low canopy burn fraction”) (Figure 1d–f).

### Study system

We studied post‐fire forest recovery in yellow pine‐mixed conifer (YPMC) forests of the northern Sierra Nevada and southern Cascade Range in California (Safford & Stevens, 2017). The region experiences a Mediterranean‐type climate with wet winters and dry summers. Averaged across our study plots, the normal mean annual temperature is 9.8°C and the normal annual precipitation is 146 cm (1991–2020 period; PRISM Climate Group, 2025). The water year following the Caldor and Dixie fires (October 2021–September 2022) was warmer and substantially drier than average, with a mean temperature of 10.8°C and total precipitation of 110 cm (PRISM Climate Group, 2025).

YPMC forests are generally dominated by ponderosa pine (*Pinus ponderosa*), white fir (*Abies concolor*), incense cedar (*Calocedrus decurrens*), sugar pine (*Pinus lambertiana*), Jeffrey pine (*Pinus jeffreyi*), Douglas‐fir (*Pseudotsuga menziesii*), and black oak (*Quercus kelloggii*). These species have no persistent soil seedbank, are not serotinous, and, with the exception of black oak, do not resprout following top‐kill. These species exhibit substantial interannual variability in reproductive output, with roughly 3–15 years between significant cone crops (“mast years”) (depending on the species) and strong intraspecific synchrony at the <1‐km spatial scale relevant to seed dispersal (Griffis & Lippitt, 2020). Pines, which are generally fire‐tolerant and shade‐intolerant, are often a special focus of forest ecology and restoration in this system given their historical dominance in many areas combined with 19th–21st century shifts away from pine dominance due to fire suppression (see next paragraph) and logging (Safford & Stevens, 2017).

The forests in our study sites historically experienced frequent fires (mean interval: 11–16 years) of generally low‐to‐moderate severity (Safford & Stevens, 2017)—in line with dry conifer forests across western North America (Agee, 1993; Fulé et al., 1997). Across California YPMC forests, a century of fire suppression has reduced the annual area burned by about eightfold (Williams et al., 2023); allowed tree density, particularly of shade‐tolerant species such as white fir and incense cedar, to increase by two‐ to fourfold (Safford & Stevens, 2017); and increased average fuel loads which, in combination with climate warming, have caused both proportional and absolute area burning at high severity to increase by roughly three‐ to sevenfold relative to the historical average (Williams et al., 2023).

### Site selection

We identified focal study areas by manually inspecting GIS data layers to locate burned areas within YPMC forests (Safford & Stevens, 2017) on USDA Forest Service (USFS) land, within approx. 1 km of accessible roads, and in areas with <30% slope. We identified YPMC forests using the USFS existing vegetation (EVEG) dataset (USDA Forest Service, 2022a), accessible roads using digitized USFS National Forest visitor maps corroborated by Google Earth imagery, USFS land using a geospatial ownership dataset (USDA Forest Service, 2022b), and slope by computing it from a 30‐m digital elevation model (USGS, 2022) using the “terra” library (Hijmans et al., 2023) in R version 4.5 (R Core Team, 2025). Within accessible YPMC forests, we located probable large high‐severity patches as those where 3‐m resolution PlanetScope satellite imagery (Planet Team, 2022) acquired between November 2021 and June 2022 contained no green but where Google Earth imagery (acquired pre‐fire) showed mature conifer forest. We located probable edge (sharp boundary between high‐severity and low‐severity) areas similarly, based on PlanetScope imagery indicating a sharp transition between green and non‐green cover. We excluded edge areas on the north side of surviving trees to minimize the effects of shading by the surviving trees on the establishing seedlings. In the field, we visited the GIS‐identified focal areas to confirm accessibility, productive mid‐ to late‐successional YPMC forest type, absence of post‐fire management, expected burn severity (i.e., 100% mortality for interior areas or sharp transition from near‐zero to near‐complete mortality for edge areas), and evidence of reproductivity of the pines (i.e., pine cones present beneath canopy‐dominant pines). We aimed to locate and sample edge areas as close as possible to the sampled interior areas (Figure 1b–d).

Within qualifying areas, we defined edge plots as those (1) with <20% of pre‐fire green canopy remaining within 10 m of plot center, (2) located as close as possible to a contiguous patch of >50 surviving green trees with >50% pre‐fire green canopy remaining (maximum distance: 60 m), and (3) with at least 10% pines (*Pinus* sp.) (by canopy volume) among the nearby surviving edge trees due to their ecological importance but relative rarity. This final criterion was allowed to be violated for a minority of edge plots (5 out of 61), which either had no pine (one plot) or 5% pine (four plots) among the nearby surviving trees. The maximum distance of 60 m from green pre‐fire canopy was selected based on previous work reporting substantially lower conifer seedling density beyond this threshold (Welch et al., 2016). The other thresholds were selected a priori with the intent of conservatively capturing severely burned plots with a high probability of seed input from nearby conifers, including pine.

For the all‐species analyses, we defined interior plots as those >60 m from the nearest surviving tree (≥5% green canopy remaining) and with no green canopy remaining within 50 m of plot center (trees with <5% green canopy remaining are very likely to die completely in 1–2 additional years; Cansler et al., 2020; Hood et al., 2007). We preferentially selected interior plots with a pre‐fire species composition that included at least 10% pines (by basal area) within 50 m, based on ocular estimates of species composition in the field. Of the 20 surveyed areas, only three areas included plots with <10% pre‐fire pine proportion, and these low‐pine plots never constituted more than half of the plots in an area.

Because of the strong ecological and forest management interest in pine restoration (see *Study system*), we performed a second set of analyses in which we only considered the regeneration of pine seedlings (thus differentiating our “all conifers” and “pine” models and results). For the pine‐specific analyses, we defined interior plots as those for which there were no surviving pine trees (i.e., ≥5% green canopy remaining) within 60 m of plot center (green canopy of other species was allowed) and no green canopy (of any species) remaining within 50 m of plot center; we additionally required the pre‐fire overstory within 50 m of plot center to contain >20% pines (by basal area). For edge plots in the pine‐specific analyses, we required that the adjacent surviving trees contain >20% pines (by canopy volume).

To evaluate the sensitivity of our inferences to the chosen minimum distance to the nearest surviving conspecific tree (i.e., 60 m), we repeated the analyses using a more conservative distance of 100 m. We report results based on the 60‐m radius here and the 100‐m radius in the appendix for comparison (Appendix S1: Tables S3–S5, Figures S7–S11). To identify the 50‐, 60‐, and 100‐m circular boundaries used for exclusion of green trees, we used a laser rangefinder (TruPulse 200, Laser Tech, Inc.) set to measure horizontal distance. We additionally measured the “sight line” as the horizontal distance to the nearest obstruction (such as a hill or dense cluster of dead trees) that might obscure the presence of a tree beyond it. We excluded interior plots with a sight line <60 m (and <100 m for the more conservative analysis reported in Appendix S1).

We sought to relatively evenly sample interior plots beneath canopy with “low canopy burn fraction” (i.e., foliage largely retained but killed and brown; Figure 1d,e) and beneath canopy with “high canopy burn fraction” (i.e., foliage largely consumed; Figure 1d,f). We generally aimed to sample about five plots per focal area, but this number varied among areas due to logistical constraints. Plots were not allowed to be within 30 m of a maintained road, to have >25% surface cover unavailable for tree seedling establishment (e.g., rock or coarse woody debris cover), or to contain non‐tree vegetation cover so dense as to potentially obscure a clear view of tree seedlings in >25% of the plot (very rare).

### Field data collection

At each plot, we recorded the number of tree seedlings by species within a variable‐size plot to ensure adequate sampling where seedlings were sparse. We determined plot size as follows (separately for each species): Begin in the NE quadrant of a circular plot with a 4‐m radius. If <10 seedlings are found, expand to the entire circular plot with a 4‐m radius. If still <10 seedlings are found, expand to a 6‐m radius. If <3 seedlings are found, expand to an 8‐m radius. If still <3 seedlings are found, expand to a 10‐m radius. Within the determined plot area, tally all seedlings of the species. Dense clusters of two or more seedlings obviously resulting from an animal cache were not included in the tally. At each plot, we visually estimated the percent cover of litter within 8 m of the plot center. Because surveys were conducted during the first growing season following the fire, litter vastly represented dropped fire‐scorched foliage. We additionally calculated “non‐growing cover” as the percentage of area within 8 m of the plot center covered by large woody debris on the ground and rocks >10 cm in the short dimension.

We also recorded the number of conifer cones, by species, on the ground within each plot using an 8‐m radius circular plot. All cones were assumed to have been dropped following the fire, as dry, opened cones from the previous season would have been consumed by fire in the high‐severity sites where we placed our plots. We also recorded an assessment of the reproductivity of the nearest canopy‐dominant tree, by species, for pines and Douglas‐fir. Specifically, we recorded “low” if there were <10 conspecific cones (except <5 for sugar pine) underneath the tree, and “high” otherwise.

We quantified canopy conditions, fire intensity, and tree mortality in and near each plot using several methods. Within a 50‐m radius of each plot center, we visually estimated the proportion of pre‐fire live canopy foliage which, at the time of survey, was (1) still green, (2) killed but retained on branches (scorched), and (3) killed and consumed (torched) or dropped. The pre‐fire live canopy foliage volume was estimated based on the presence of fine branches. Although pre‐fire live branches often remain following fire, they may be consumed by very high‐intensity fire. Therefore, our estimate of the proportion of pre‐fire canopy volume that was scorched and torched may be negatively biased for the highest fire intensities, but it should still increase monotonically with fire intensity. At each plot, we also conducted a rapid visual estimate of the pre‐fire overstory species composition, by basal area, within a 50‐m radius of each plot center. At edge plots, we similarly estimated the species composition of the adjacent contiguous patch of surviving trees as well as the distance from plot center to the patch edge.

### Plot‐level data preparation

At all plots, we quantified fire intensity as “canopy burn fraction” by subtracting from 100 the average of the percent litter cover (in each 8‐m plot) and the percent of scorched canopy foliage (within 50 m of plot center). That is,

(1) $$p_{\text{canopy}\_\text{burn}}=100-\left(p_{\text{litter}}+p_{\text{scorched}}\right)/2,$$

where *p*_canopy_burn_ is the canopy burn fraction, *p*_litter_ is the percent litter cover, and *p*_scorched_ is the estimated percent of pre‐fire green canopy foliage that was killed but retained in the canopy at the time of survey. We then classified plots as “high canopy burn fraction” if the torching extent was greater than 85% and “low canopy burn fraction” otherwise. This yielded a dataset of 45 low canopy burn fraction interior plots, 51 high canopy burn fraction interior plots, and 61 edge plots.

We quantified seedling density in the plots, by species, as the number of observed seedlings divided by the area of the plot in which the seedlings were tallied, adjusted based on the non‐growing cover. Specifically,

(2) $$d_{\text{seedlings}}=c_{\text{seedlings}}/\left(a_{\text{plot}}\times \left(1-c_{\text{non}-\text{growing}}\right)\right),$$

where *d*_seedlings_ is the seedling density (in seedlings per square meter), *c*_seedlings_ is the seedling count, *a*_plot_ is the area of the plot (in square meters) in which the seedlings were tallied, and *c*_non‐growing_ is the percent cover of non‐growing area in the plot. The purpose of this correction was to account for plot area that could not support seedlings and would thus decouple observed seedling counts from the quantity of deposited seeds.

### Day of burning

We obtained day of burning estimates for our study plots, across our study fire footprints, and across all California fires burning between 2002 and 2020 using interpolated satellite hotspot detections following the method presented in Parks (2014) (Appendix S1, Section S1: Supplemental methods).

### Expected decline in seed deposition beyond the green forest edge

To compare our field observations against a counterfactual expectation of seeds originating only from surviving trees, we calculated the expected decline in seed deposition from a green forest edge using the micrometeorological dispersal equation of Greene and Johnson (1996) with a wind regime appropriate for leafless forests (their eq. 22). Terminal velocities for the three primary conifers in this paper (white fir, ponderosa pine, and Jeffrey pine) were derived from their mean seed masses via Greene and Johnson (1993; their equation in tab. 3 for Pinaceae).

### Characterizations of sampled gradients

To provide context in interpreting results, we created a series of scatterplots depicting distance to edge versus day of burning (Appendix S1: Figure S3) and distance to edge versus mean annual precipitation (Appendix S1: Figure S4) for edge plots, and mean annual precipitation versus day of burning for all plots (Appendix S1: Figure S5). To characterize the climate of our plots, we extracted normal annual precipitation (1981–2010 mean) from the 800‐m resolution PRISM dataset (PRISM Climate Group, 2025) using bilinear interpolation.

### Summary statistics

Because of the highly right‐skewed seedling density distribution, we summarize observed seedling densities as the median density across plots of a given type. We also report species composition by computing the median seedling density, by species, for the given plot type, and then relativizing those median densities so they sum to 100%. This is an alternative to relativizing mean densities and is less sensitive to rare extremely large seedling counts. However, it causes species present in <50% of plots (median density: 0) to represent 0% of the computed species composition.

### Statistical analysis

We fitted generalized additive models (GAMs) to explain seedling density and quantify its relationship with environmental predictors. We fitted four separate models: one for each factorial combination of species group (all or pines) and plot type (edge or interior). Models used the plot as the observation and included the smoothed predictor terms precipitation, canopy burn fraction, and (for the pine models only) the pre‐fire overstory pine proportion (for the interior model) or the green edge pine proportion (for the edge model). We fitted GAMs using cubic splines, and to constrain the fits to have at most one curve, we specified a maximum basis dimension of 3. We estimated smoothing parameters using restricted maximum likelihood (REML). We represented seedling density as the expected count of seedlings within a 10‐m radius plot (computed based on the recorded seedling density) and modeled errors using a negative binomial distribution. We excluded plots burning prior to 1 August from the GAMs due to the inferred absence of viable canopy seeds at the time of fire (see *Results* section “Fire timing,” below). For the edge plot models, we included only plots within 30 m of the green edge (*n* = 28 for all species and 23 for pines) in order to focus on plots with the greatest green edge effect. To quantify the importance of fire intensity, we fitted an additional set of models excluding the canopy burn fraction term and quantified the variation explained by the fire intensity term as the difference in deviance explained between the full model and the reduced model.

To visualize model fits, we created scenario figures in which we predicted observations along a continuum of one predictor (torching extent or precipitation), holding all other predictors constant at their medians or at alternative values as specified in the figure captions. We plotted predictions between the 2.5th and 97.5th percentiles of the predictor variable (*x*‐axis of scenario figures). Although we do not have specific study questions around precipitation, we include it as a covariate because it can explain substantial variation in seedling establishment (Stewart et al., 2021) and thus accounting for it aids in interpreting the effects of the other predictors of interest. We fitted, inspected, and predicted from GAMs using the mgcv package (Wood, 2011) in R version 4.5 (R Core Team, 2025).

## RESULTS

### High seedling densities in the absence of surviving trees

**FIGURE 2:**
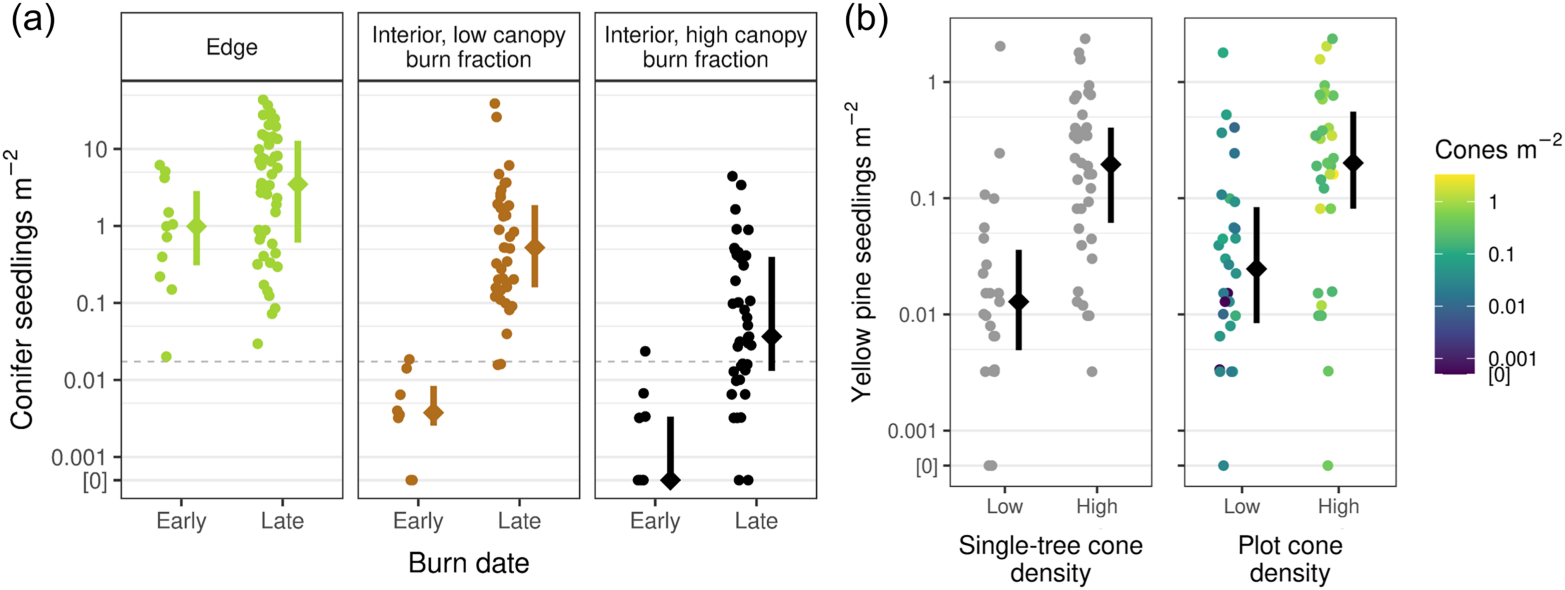
Observed seedling densities relative to potential canopy seed survival predictors. (a) Observed seedling densities versus burn date (one point per plot, colored by plot type). Edge plots were <60 m from surviving reproductive trees, while interior plots were >60 m. “Early” burned plots burned before 1 August, while “late” burned plots burned after. The dashed horizontal line indicates a regional reforestation target of 175 seedlings ha^−1^ (Welch et al., 2016). (b) Yellow pine seedling density versus conspecific cone count under the nearest overstory conspecific tree (left panel; “high” indicates ≥10 cones) and cone density within the seedling plot (right panel; “high” indicates density greater than the plot‐level median density of 1490 cones ha^−1^) for plots that burned in August or later. Points represent plots. Within each plot category, the vertical bar spans the interquartile range of observed seedling density and the diamond indicates the median.

Natural tree regeneration in high‐severity fire interiors generally far exceeded levels needed to support forest recovery, despite the lack of surviving trees (Appendix S1: Table S1). Across all high‐severity interior plots, the median conifer seedling density was 1040 trees ha^−1^. This density greatly exceeds the average historical (pre‐fire suppression) tree density in these forests of approximately 150 trees ha^−1^ (Safford & Stevens, 2017) and a regional target for post‐fire reforestation of 175 seedlings ha^−1^ (Welch et al., 2016) (“replacement density”). The densities we observed were far greater than expected if the seeds originated only from surviving trees on the landscape (as determined via our counterfactual exploration; Appendix S1: Table S2). Seedling densities in interior plots sometimes approached the densities in edge plots (Figure 2a), where high levels of seed deposition are traditionally expected due to the proximity of surviving trees. Further, yellow pine (*Pi. ponderosa* and *Pi. jeffreyi*) seedling density in interior plots was eight times greater where conspecific cones were abundant than where they were scarce (Figure 2b). Although the seedling pool was primarily composed of yellow pines and white fir (see *Species composition*, below), white fir, Douglas‐fir, sugar pine, and yellow pines all had seedling densities >1000 seedlings ha^−1^ in at least one interior plot.

### Fire timing

Seedling densities in interior plots generally exceeded replacement density and approached densities in nearby edge plots, with an important exception: plots that burned prior to August (median seedling density: 32 seedlings ha^−1^) (Figure 2a; Appendix S1: Figure S6). In these early‐burned areas, the edge plot seedling densities remained high (median: 9900 seedlings ha^−1^), indicating that conditions were generally favorable for seed production and seedling establishment the year after fire. Focusing on interior plots that burned after 1 August, the median density was 2010 seedlings ha^−1^. Our analyses of wildfire progression data revealed that between the years 2002 and 2020, 72% of burned area in California burned between 1 August and 31 October—the period in which seeds of mixed‐conifer species generally ripen (Griffis & Lippitt, 2020)—as did 73% of our study fire footprints.

### Fire intensity

**FIGURE 3:**
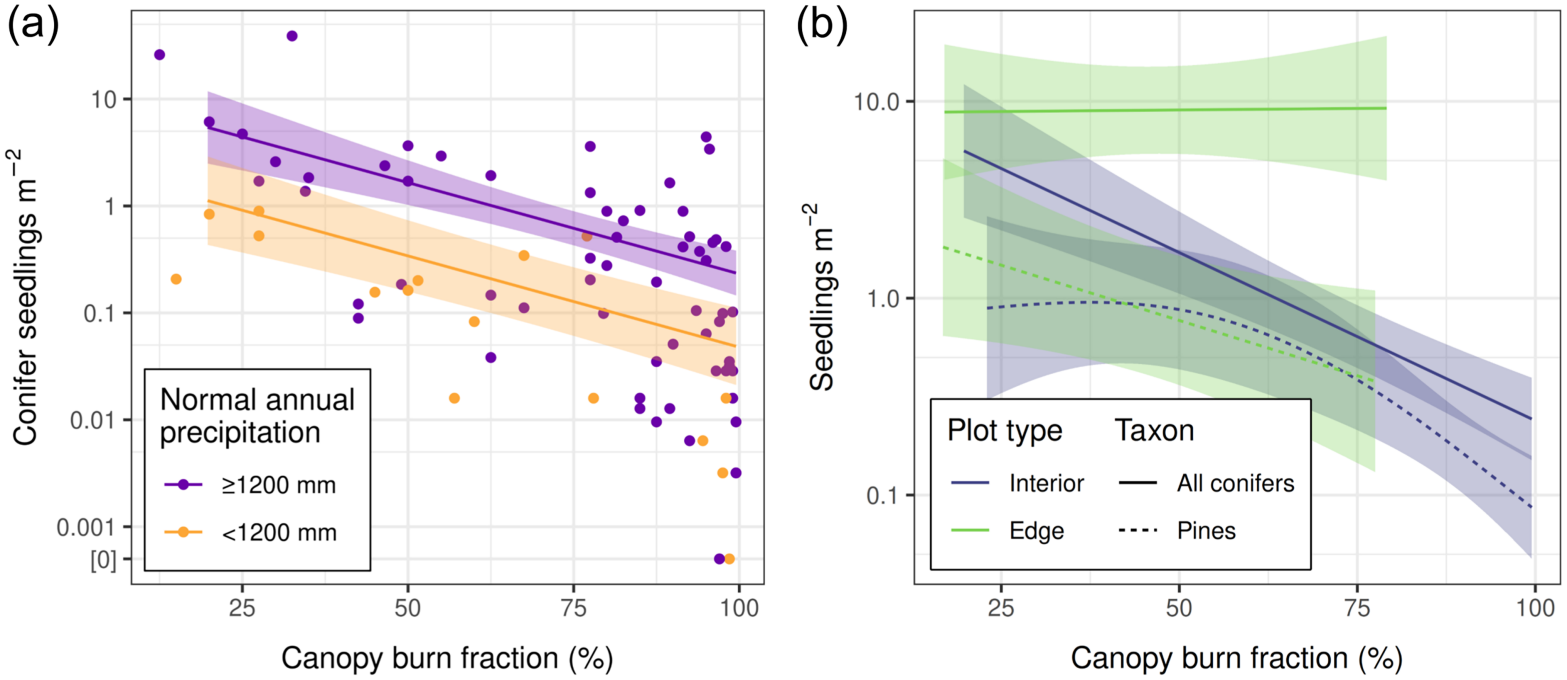
Model‐fitted seedling density (lines) versus canopy burn fraction for different plot subsets. Panel (a) depicts fitted density of all conifer seedlings for all later‐burned high‐severity interior plots >60 m from surviving conspecific trees. The panel includes raw data (one point per plot) and differentiates plots by high and low precipitation (colors), with model fits assuming the median precipitation within each class. Two plots with a seedling count of exactly zero are displayed on the line labeled “[0],” discontinuous with the remainder of the axis. Panel (b) depicts the fits of four separate models for different plot types (interior and edge; colors) and taxonomic groups (all conifers and pines only; line styles). In both panels, model predictors besides canopy burn fraction (and for panel (a), normal annual precipitation) were held at their means. The precipitation effect was included because it is an influential covariate important to account for when interpreting the effect of canopy burn fraction. The continuous fit of the precipitation term in these models is depicted in Appendix S1: Figure S2.

We found that within high‐severity (100% mortality) patches, seedling density was significantly and substantially explained by the estimated proportion of foliage consumed by fire—a potential surrogate for fire intensity within the crown (Hood et al., 2018). Along a continuum from 25% to 95% canopy burn fraction, model‐fitted interior plot seedling densities decreased by about 16‐fold (Figure 3); canopy burn fraction explained 32% of the deviance in seedling density in later‐burned interior plots (Table 1). Pines, specifically, were less sensitive to this fire intensity surrogate, with model‐fitted seedling densities declining 7.5‐fold along the same canopy foliage consumption continuum (Figure 3b). Canopy burn fraction explained only 22% of the deviance in pine seedling density (Table 1). Canopy burn fraction was a far less important predictor of seedling density in edge plots, explaining only 0.2% of deviance in seedling density (Table 1). However, for pines specifically, canopy burn fraction was moderately important, explaining 9.7% of deviance (Table 1)—still far less than in interior plots.

**TABLE 1:** Generalized additive model (GAM) metrics indicating that fire intensity explains substantial variation in seedling density in interior plots and much less in edge plots.

Plot type	Species group	Sample size (plots)	Full model effective degrees of freedom	Deviance explained (%)		
				Full model	Full model minus “canopy burn fraction”	“Canopy burn fraction”
Interior	All conifers	79	3.37	41.8	10.3	31.5
	Pines	65	5.61	23.0	1.1	21.9
Edge	All conifers	28	3.43	23.3	23.1	0.2
	Pines	23	5.26	40.5	30.8	9.7

*Note*: The “All conifers” full model includes the smooth terms “precipitation” and “fire intensity” and the “Pines” full model includes these terms plus “pre‐fire overstory pine proportion” (for interior plots) or “seed wall overstory pine proportion” (for seed wall plots). The “interior” models include only plots >60 m from surviving reproductive trees, the “edge” models include only plots <30 m from surviving trees, and all models exclude plots that burned prior to 1 August.

### Species composition

Regenerating seedlings in later‐burned interior plots were overwhelmingly dominated by yellow pines (81%), with the remainder of seedlings representing white fir (19%). In contrast, edge plot seedlings were dominated by white fir (91%), with the remaining seedlings representing yellow pines (7%) and incense cedar (2%). Although other species—particularly Douglas‐fir and sugar pine—were present, they are not represented in these summaries, which were obtained by relativizing plot‐level median species densities; all other species had a median density of 0. Species composition also depended on local fire intensity: White fir seedlings were far more prevalent in later‐burned interior plots at low (0%–50%) canopy burn fraction (76% white fir and 24% yellow pine) than at high (80%–100%) canopy burn fraction (27% white fir and 73% yellow pine).

Because pines are now relatively less abundant, we intentionally stratified plots into areas that contained pine adults prior to fire, potentially also inflating pine seedling density relative to average expectations across a landscape. Nonetheless, the interior plot seedling density was far more dominated by pines than was the overstory of these same plots (mean canopy composition: 42% yellow pines, 36% white fir, 12% incense cedar, 7% sugar pine, and 2% Douglas‐fir).

## DISCUSSION

### Evidence for canopy seed survival

Multiple lines of evidence support the interpretation that the high densities of seedlings we observed in interior plots, with no surviving trees nearby, originated from seeds that survived in the canopies of fire‐killed trees. First, the observed seedling densities (median: 1040 trees ha^−1^ across all interior plots; 2010 trees ha^−1^ in later‐burned interior plots) were far greater than would be expected if all the seedlings originated from seeds dispersed from the nearest surviving trees (Appendix S1: Table S2). In previous studies of high‐severity areas where canopy seed survival has not been documented, expected seedling densities often, but not always, drop to near zero beyond 75–100 m from surviving trees (Bonnet et al., 2005; Chambers et al., 2016; Davis et al., 2019; Kemp et al., 2016; Owen et al., 2017; Ritchie & Knapp, 2014; Rother & Veblen, 2016; Appendix S1: Figure S1). A second line of evidence supporting canopy seed survival is the observation that yellow pine seedling densities were eight times greater in plots where yellow pine cones were abundant than elsewhere (Figure 2b), strongly suggesting that seeds originated predominantly from local, reproductive trees killed by fire, rather than distant surviving trees.

A third line of evidence of a local fire‐killed seed source relates to our observation of a strong effect of fire timing. The fact that seedling densities remained high in early‐burned edge plots but not in early‐burned interior plots implies that abiotic conditions were suitable for seedling establishment, but that ripe seeds were simply not yet available in interior plots at the time of fire. Seeds from this study's focal tree species do not typically begin to ripen until early August each year (Griffis & Lippitt, 2020; Lopez et al., 2025), and thus the presence of high‐intensity fire prior to 1 August likely interrupted the seed ripening process and significantly reduced the availability of viable seed post‐fire. On the other hand, seeds of surviving trees adjacent to edge plots were allowed to continue to ripen following fire and later disperse into edge plots. This evidence implies that for interior plot seedling densities to be high, as we observed in later‐burned areas, the timing of fire must overlap with the timing of seed ripeness.

A fourth line of evidence for local seed origin is the strong correspondence—in later‐burned interior plots—between seedling density and foliage consumption (canopy burn fraction), a surrogate for fire intensity. Even under high fire severity (e.g., 100% tree mortality), fire intensity (heat output and flame lengths) can vary (Mercer et al., 1994). Simulations and experiments have shown that with sufficient temperature and residence time, fire can kill seeds within cones (Lopez et al., 2025; Mercer et al., 1994; Michaletz et al., 2013). In our study, we found lower seedling densities in later‐burned interior areas with higher foliage consumption, implying that higher intensity fire increases the likelihood of seed mortality in tree canopies. The much weaker observed relationship between fire intensity and seedling density in edge plots supports the interpretation that, in contrast to interior plots, edge plot seedlings originated largely from adjacent surviving trees.

One may posit that the negative relationship between seedling density and canopy burn fraction observed in interior plots could be driven by site suitability factors associated with fire intensity (e.g., shading or mulching by fire‐killed needles; Bonnet et al., 2005), rather than by variation in canopy seed survival. However, the fact that the association between fire intensity and seedling densities was much weaker in edge plots than in interior plots suggests that fire intensity‐associated site suitability does not explain most of the variation in seedling density.

Secondary animal dispersal of our primarily wind‐dispersed study species may extend their dispersal distances (Vander Wall, 2003), potentially enabling transport of seeds into severely burned interiors from distant surviving trees. However, dispersal by rodents—the predominant secondary dispersers in our study system—is usually very modest (<20 m; Cao et al., 2016; Dou et al., 2024; Han et al., 2025). More rarely, birds may disperse small numbers of seeds >1 km (Vander Wall, 2023). No work to our knowledge has specifically quantified secondary dispersal in recently burned dry conifer forests, though we believe its influence on our observations is minimal because (1) our seedling counts intentionally excluded seedlings obviously resulting from rodent and bird caches (clumps of two or more seedlings; though single‐seed caches are possible; Vander Wall, 2023); (2) any secondary dispersal effect should also be captured by existing post‐fire regeneration studies, which generally find very little dispersal beyond 100 m from surviving trees (first paragraph in this section); and (3) three of the lines of evidence for canopy seed survival presented in this section show that seedling densities vary substantially along multiple axes of local conditions (pine cone density, canopy burn fraction, and burn date) that do not obviously relate to secondary disperser cache site selection.

Within‐plot yellow pine cone densities were substantially (roughly 2.5–3 times) higher in interior plots with low canopy burn fraction than in other areas (high burn fraction interior plots and edge plots) (Appendix S1: Table S1). We believe that this pattern is most likely driven by our sampling design: we required interior plots to have >10% pine representation in the fire‐killed canopy within 50 m of the plot, while for edge plots, we required the surviving trees closest to the plot (not the fire‐killed trees immediately surrounding the plot) to contain >10% pine. This difference in plot selection rules may have resulted in interior plots having more fire‐killed pines—and thus the cones they produce—close to and in the plot. One line of evidence suggests that such a sampling artifact is the dominant driver: the fraction of plots with a highly reproductive yellow pine individual nearby (either the closest canopy‐dominant fire‐killed tree in the case of interior plots, or surviving tree in the case of edge plots) was very consistent between interior and edge plots (Appendix S1: Table S1). Alternatively, local tree fecundity may be correlated with, and potentially driven by, factors that also control upper canopy fire intensity, such as local soil moisture and crown separation from understory fuels (Agee & Skinner, 2005; Krawchuk et al., 2016)—possibly explaining both the high seedling and cone densities in these interior areas. Whether the greater in‐plot cone density of interior plots with lower canopy burn fraction is a sampling artifact or reflects an unexplained real difference between pine reproductive output in edge versus interior plots, the higher yellow pine cone counts do not affect our main findings: that the later‐burned interior plots contained seedling densities far exceeding replacement densities, that these high densities are difficult to explain via long‐distance dispersal, and that multiple lines of evidence support the local origin of seed in interior plots (i.e., the influence of canopy burn fraction, fire timing, and local cone density within later‐burned plots).

### Canopy seed survival varies by species

Although numerous species appear to exhibit some degree of canopy seed survival through extreme fire (white fir, Douglas‐fir, sugar pine, and yellow pine all had seedling densities >1000 seedlings ha^−1^ in at least one interior plot), it appears to be particularly strong and widespread in yellow pine. Yellow pine was by far the most abundant species among seedlings in interior plots, a notable observation given that many conifer forests that were historically dominated by pines have become overwhelmingly dominated by shade‐tolerant species, including white fir, as a consequence of logging and fire suppression (Safford & Stevens, 2017). It is generally thought that low‐ to moderate‐severity fire is an effective means for maintaining or restoring overstory pine dominance in YPMC forests given the relative fire tolerance of pines, although recent work suggests that low‐intensity fire alone will not shift forest composition back toward pines (May et al., 2023). Our results suggest that when reproductive phenology and fire dates align, even high‐severity fire may, over a much longer time period, also help to promote pine restoration—at least relative to a no‐fire scenario.

The greater apparent canopy survival of pine seeds versus other species in interior plots—and the fact that interior plot pine seedling density was less sensitive to variation in fire intensity than that of other species—aligns with the documented ability of ponderosa pine seeds to survive higher heat exposure than many other species while inside closed cones (Greene et al., 2024; Lopez et al., 2025), though the heat tolerance of seeds in ponderosa pine cones relative to those of white fir specifically is unknown. The lower abundance of white fir seedlings in the interior, despite high abundance at edges, may be explained by less protected seeds or by a reproductive phenology not aligned with fire timing. In contrast to interior plots, in edge plots, pine seedling density was more sensitive to fire intensity than that of other species. This contrasting result may reflect the fact that pine seeds are heavier—and therefore limited to shorter dispersal distances—than other species in the system (Greene & Johnson, 1993; Safford & Stevens, 2017; Siggins, 1933), and thus pine seedling establishment may be more dependent on seeds arriving from (fire‐killed) trees located directly above a plot than from surviving trees adjacent to that plot.

### Prevalence of canopy seed survival

Previous suggestions of possible canopy seed survival through high‐severity fire in non‐serotinous conifers are limited, and they are constrained to forests adapted to substantial high‐severity fire (Gray & Franklin, 1997; Harris et al., 2020; Larson & Franklin, 2005; Pounden et al., 2014). Although the paucity of reports in frequent‐fire forests suggests that canopy seed survival is rare in this system, it is also possible that it is frequently missed, especially in years with low‐to‐average seed production. Many of the corroborating predictors (e.g., canopy burn fraction, cone density, and pre‐fire overstory species composition) are not easily quantified >3 years following fire, when most post‐fire surveys are conducted (Davis et al., 2023), and at that point, the influence of competing vegetation and other site suitability factors may dominate. Some previous work across extensive plot networks has found that seedling prevalence, especially for yellow pines, is relatively poorly explained using common predictors such as distance from living reproductive trees (Davis et al., 2023; Stewart et al., 2021; Young et al., 2019). Our study suggests that the previously unexplained high seedling densities occasionally observed far from surviving trees (e.g., Appendix S1: Figure S1) could be a signature of canopy seed survival.

Will canopy seed survival meaningfully boost resilience of western dry conifer forests through the 21st century? The answer depends strongly on three enabling factors: (1) alignment of fire timing and reproductive phenology, (2) cone crop volume in the year of fire, and (3) survival of the first post‐fire cohort of seedlings. First, wildfires must occur in late summer when a large crop of conifer seeds is ripe but still retained in cones. In four conifer species in northern California and across two years, the majority of seeds inside cones were observed to reach maturity between early August and mid‐September (Lopez et al., 2025). In turn, seed abscission, which typically starts in early September and continues throughout the autumn (e.g., Devine & Harrington, 2016; Dobbs, 1976; Griffis & Lippitt, 2020; Jemison & Korstian, 1944; Vander Wall, 1997), marks the end of canopy seed availability. Thus, we expect the strongest canopy seed survival in non‐serotinous conifers to be observed when stand‐replacing fire occurs within a specific temporal window, bounded on one end by seed maturation and on the other by seed abscission—under current conditions, between early August and September or October (Figure 1a). This period encompasses the burn date range of our later‐burned plots, which exhibited substantial evidence of canopy seed survival and forest regeneration far from surviving trees.

The fact that 72% of the area burned in California between the years 2002 and 2020—and 73% of the footprint of our study fires—burned between 1 August and 31 October suggests an alignment between the canopy seed ripeness window and the annual period of high fire activity. This result corresponds with the work of Lopez et al. (2025), who defined the canopy seed survival period as a narrower range of late July to mid‐September and found that roughly 60% of burned area in California burned during this period. Nonetheless, climate change may shift both fire timing and tree reproductive phenology in ways that are difficult to predict, potentially threatening the longevity of canopy seed survival as a resilience mechanism. For example, as fire seasons lengthen due to the increased prevalence of weather conducive to extreme fire (Westerling et al., 2006), a greater percentage of area may burn earlier, outside the current canopy seed survival window. However, warming may also shift reproductive phenology earlier (Gordo & Sanz, 2010), and the impact of such shifts on forest resilience via canopy seed survival hinges on whether they will occur in synchrony with fire season changes. Potentially compounding the effect of a lengthening fire season, many areas are beginning to experience shorter fire return intervals, with severely burned areas reburning within several decades (Coop et al., 2020). Recurring fire may kill many reestablishing trees (Hoecker & Turner, 2022), undermining any resilience contributed by canopy seed survival. That said, when reburns occur prior to substantial fuel accumulation, such fires may be lower severity (Tortorelli, Latimer, et al., 2024) and allow establishing saplings to survive (York et al., 2021; York & Russell, 2025).

The second factor enabling canopy seed survival to contribute meaningfully to fire resilience is the presence of a substantial seed cone crop the year of the fire. Relative to species with true serotiny—which accumulate cones with viable seeds over multiple years prior to disturbance—our study species release the bulk of their viable seeds each year. Thus, the size and quality of just the current year's seed crop strongly determine the potential for canopy seed survival to support forest recovery. Based on observed masting intervals (Griffis & Lippitt, 2020), the probability of any one of our focal conifer species having a significant cone crop in any given year averages roughly 5%–30%. Prediction of mast years more than a few months in advance can be challenging and highly species‐specific (Bogdziewicz et al., 2024), and patterns are likely to be perturbed by ongoing climate change (Bogdziewicz, 2022), constraining our ability to anticipate potential synchrony between large cone crops and wildfire.

Finally, for canopy seed survival to have lasting positive effects on forest recovery, the initial post‐fire seedling pulse must persist. The survival rates of seedlings less than 1 year old are not well known, but encouragingly, recent work in our study system shows that seedlings planted early (1–2 years post‐fire), before competing vegetation has become established, have relatively high survival rates (Sorenson et al., 2025). Relatedly, strong natural pine recruitment by the fifth year post‐fire generally predicts continued forest recovery a decade later (Tortorelli, Young, et al., 2024). For successful early seedling establishment, weather conditions in the years following fire must also be conducive (Hankin et al., 2019; Harvey et al., 2016; Young et al., 2019)—something that is not at all guaranteed and is projected to decline in probability as the climate warms and droughts become more frequent and severe (Davis et al., 2019). However, the fact that we observed substantial regeneration despite the relatively warm, dry post‐fire year suggests that the seasonal distribution of precipitation (e.g., duration of the summer dry season) is also important for seedling establishment, potentially enabling forest recovery in some years even as aridity increases overall.

### Management considerations and conclusion

Given current reforestation capacity constraints (North et al., 2019), an improved ability to predict when and where natural post‐fire regeneration will be strong could increase management efficiency by enabling reforestation efforts to be more precisely prioritized in the areas where they are most needed. In years and locations where the factors enabling canopy seed survival (fire timing in relation to reproductive phenology, masting, and post‐fire weather) align, there may be less need for active reforestation within high‐severity interiors—particularly those areas escaping the most extreme fire (i.e., where some scorched foliage remains). Relative to traditional post‐fire natural regeneration that is often dominated by shade‐tolerant species able to establish over years to decades under a shrub canopy (Lauvaux et al., 2016; Tubbesing et al., 2020), the regeneration immediately following fire in areas with canopy seed survival may be enriched with the historically dominant pines, further highlighting the potential assistance that canopy seed survival may provide in the restoration of historical forest conditions.

Nonetheless, the high densities of standing (and eventually falling) dead trees (“snags”) in many severely burned areas may create challenges for long‐term maintenance of natural regeneration (e.g., thinning to achieve target densities) both by constraining site access and by serving as fuel for future high‐severity fires that could eliminate the establishing cohort. Preemptive mitigation of these challenges through post‐fire snag removal may be counterproductive, as machinery may crush a substantial fraction of tree seedlings (Donato et al., 2006; but see Povak et al., 2020). If snags are a concern, one solution may be relatively rapid reintroduction of fire during mild fire weather, with the goal of consuming some of the fuels and competing vegetation while allowing many of the establishing trees to survive (York et al., 2021; York & Russell, 2025).

The possibility that canopy seed survival may lead to successful natural forest regeneration provides an additional motivation for pre‐fire management such as stand thinning and prescribed or cultural burning to reduce fuel loads and tree densities. Even if these treatments fail to moderate fire severity and nearly all overstory trees are killed in wildfire, fuel reduction may result in lower fire intensity that improves seed survival in cones. Further, lower‐density stands that ultimately burn at high severity would produce fewer snags to impede future maintenance of natural regeneration.

Given the numerous complex and intersecting factors required for canopy seed survival to result in meaningful recovery, it is probably the exception rather than the norm. However, in some important cases, it appears to support substantially greater forest resilience to high‐severity fire than current models predict.

## AUTHOR CONTRIBUTIONS

*Conceptualization*: Derek J. N. Young, David F. Greene, and Andrew M. Latimer. *Methodology*: Derek J. N. Young, David F. Greene, Andrew M. Latimer, and Nina E. Venuti. *Data collection*: Derek J. N. Young, Nina E. Venuti, and Andrew M. Latimer. *Data analysis*: Derek J. N. Young, David F. Greene, and Andrew M. Latimer; *Visualization*: Derek J. N. Young. *Funding acquisition*: Derek J. N. Young and Andrew M. Latimer. *Project administration*: Derek J. N. Young. *Supervision*: Derek J. N. Young. *Writing—original draft*: Derek J. N. Young, Andrew M. Latimer, David F. Greene, and Nina E. Venuti. *Writing—review and editing*: Derek J. N. Young, Andrew M. Latimer, David F. Greene, and Nina E. Venuti.

## CONFLICT OF INTEREST STATEMENT

The authors declare no conflicts of interest.

## Supporting information


Appendix S1.

## Data Availability

The field plot data and day of burning data (Young et al., 2025) are available in Dryad at https://doi.org/10.25338/B8D07V. The code for preparing, analyzing, and visualizing data (Young, 2025) is available in Zenodo at https://doi.org/10.5281/zenodo.16427753.
