# Supplementary material for: Canopy seed survival through extreme fire in non‐serotinous conifers: An unexpected source of forest resilience
Source: Ecol Appl. 2025 Nov 17;35(7):e70142. doi: 10.1002/eap.70142 (PMC12623069; doi:10.1002/eap.70142)
Supplement: Supplementary file 1 — Appendix S1. [file EAP-35-e70142-s001.pdf]

Appendix S1 for

Canopy seed survival through extreme fire in non-serotinous conifers: An unexpected source of forest resilience

Ecological Applications

Derek J. N. Young, Nina E. Venuti, David F. Greene, Andrew M. Latimer

This PDF file includes:

Section S1: Supplemental methods
Section S2: Figures S1 to S11
Section S3: Tables S1 to S5
Section S4: Appendix S1 references

Section S1: Supplemental methods

Estimating day of burning

We mapped day of burning at 30 m resolution for the Caldor and Dixie fires by interpolating satellite hotspot detections following the method presented in Parks (2014), incorporating hotspots detected by the MODIS (Moderate Resolution Imaging Spectroradiometer) and VIIRS (Visible Infrared Imaging Radiometer Suite) sensors as provided by the NASA Fire Information for Resource Management System (FIRMS) (Davies et al. 2019). We extracted the resulting day of burning value at each of our field plots and classified plots into "early burning" (burned prior to 1 August) or "later-burning" (burned on or after 1 August). All of our plots burned prior to 15 September.

For assessment of fire seasonality, we additionally used progression maps for the California fires burning between 2002 and 2020, produced using the same methods, as published in Coop et al. 2022 and provided by S. Parks. We combined these with day of burning maps that we calculated, using the same methods, for California fires > 404 ha burning in 2021 based on perimeters from the Monitoring Trends in Burn Severity (MTBS) database (Eidenshink et al. 2007). We then summarized fire seasonality by calculating the proportion of pixels burning between 1 August and 31 October across a given spatial domain (California, or Caldor and Dixie fires). This is the period in which seeds of mixed-conifer species generally ripen in California (Griffis and Lippit 2020) and it encompasses our "later burned" plot category.

Section S2: Supplemental figures

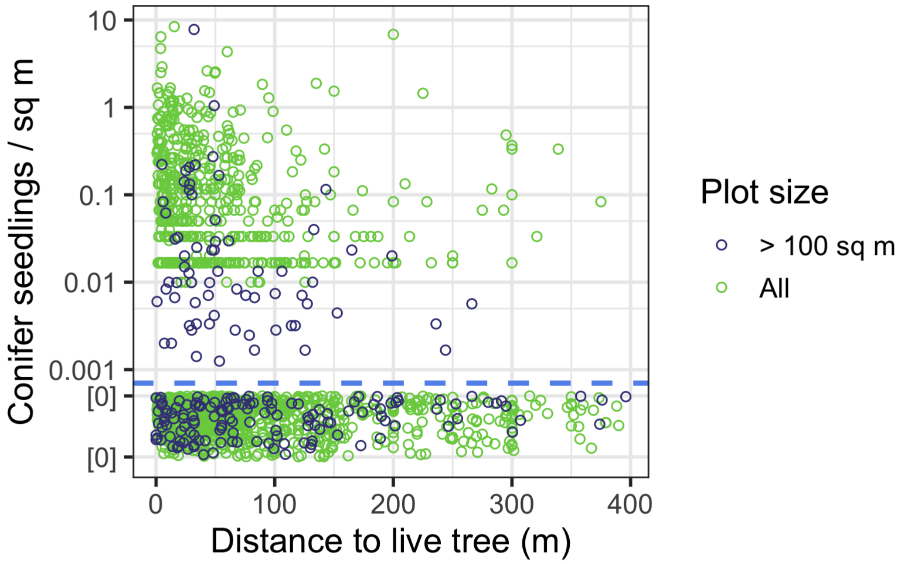

Fig. S1. Conifer seedling density as a function of distance to the nearest live tree from a published compilation of > 10,000 post-fire regeneration plots from across the western United States (Davis et al. 2023), subset to plots that burned at high severity (RBR > 300) and excluding lodgepole pine (Pinus contorta) (and plots within the distribution of P. contorta but no other focal conifers) because the species is often serotinous and thus often does not rely on surviving trees for reproduction. Plots with seedling counts of exactly zero are displayed as a band of vertically-jittered points between the tick marks labeled "[0]" below the dashed blue line, discontinuous with the remainder of the axis, which uses a log scale between 0.001 and 10 seedlings m-2. For comparison, recommendations for post-fire reforestation seedling densities in the region range from 0.0124 to 0.0741 seedlings m-2 (North et al. 2019).

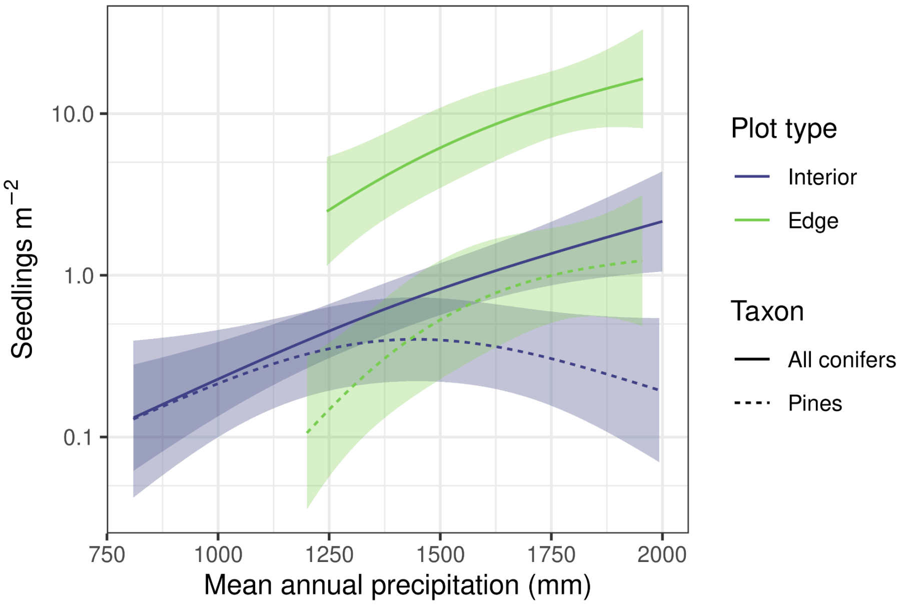

Fig. S2. The fits of the four separate GAMs for different plot types (interior and edge) and species groups (all conifers and pines only; colors) relative to mean annual precipitation, with all other model predictors held at their means.

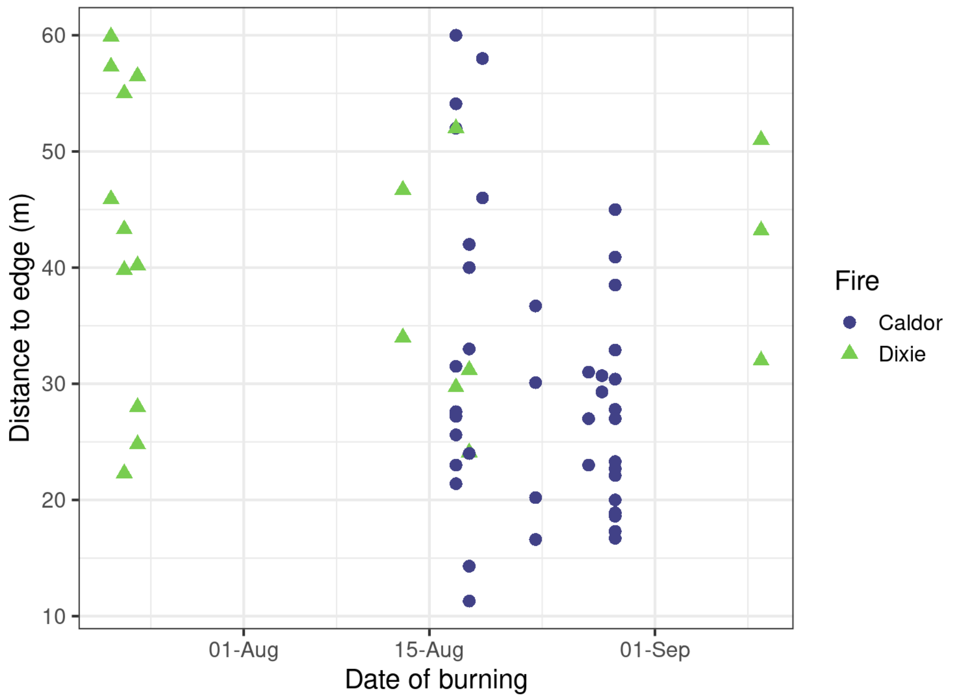

Fig. S3. Distance to green forest edge vs. day of burning for edge plots (points) included in the all-species analysis.

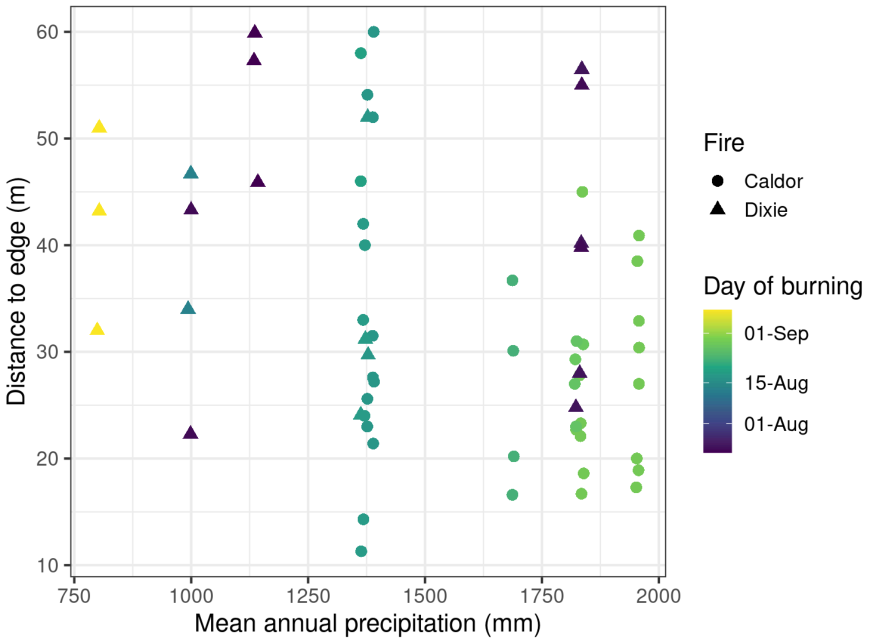

Fig. S4. Distance to green forest edge vs. mean annual precipitation (with points colored by day of burning) for edge plots (points) included in the all-species analysis.

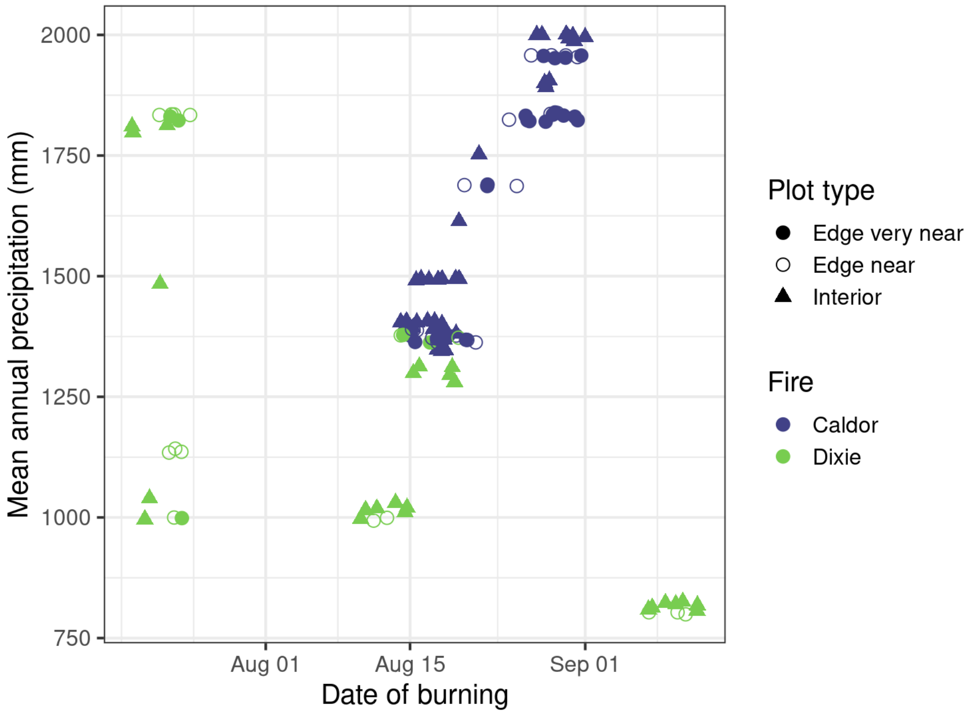

Fig. S5. Mean annual precipitation vs. date of burning for plots (points) included in the all-species analysis. Points are jittered ± 3 days on the x-axis to increase visibility.

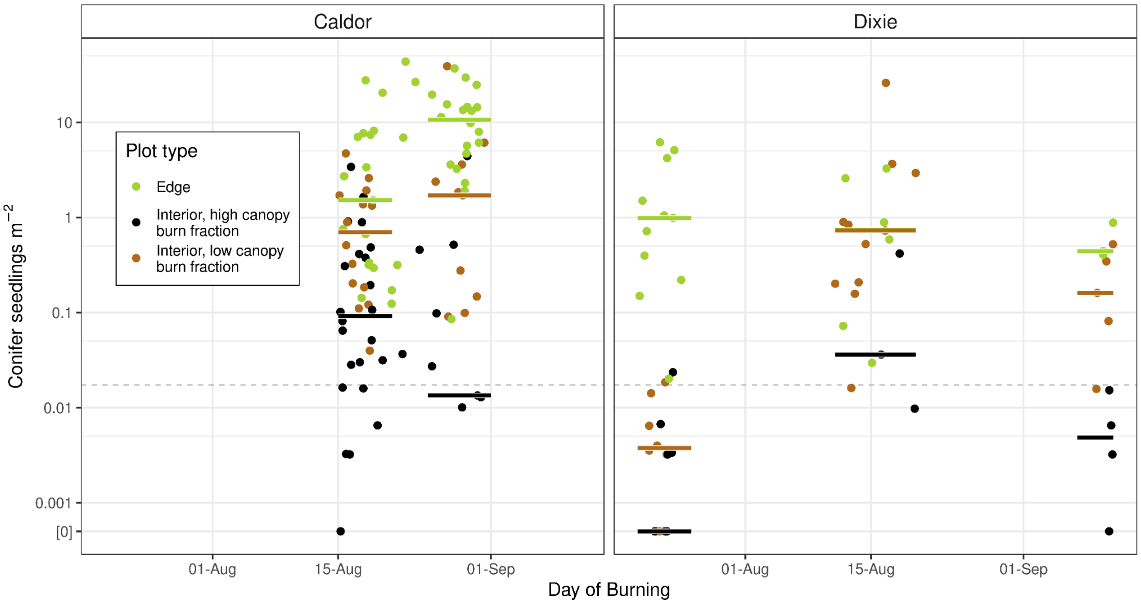

Fig. S6. Observed seedling densities versus burn date (one point per plot, colored by plot type). Edge plots were < 60 m from surviving reproductive trees, while interior plots were > 60 m. Points are jittered ± 2 days on the x-axis to increase visibility. Thick horizontal lines indicate median densities by plot type (colors; both edge plot types combined) within several date ranges (horizontal line extent). The dashed horizontal line indicates a regional reforestation target of 0.018 seedlings·m^{-2} (Welch et al. 2016).

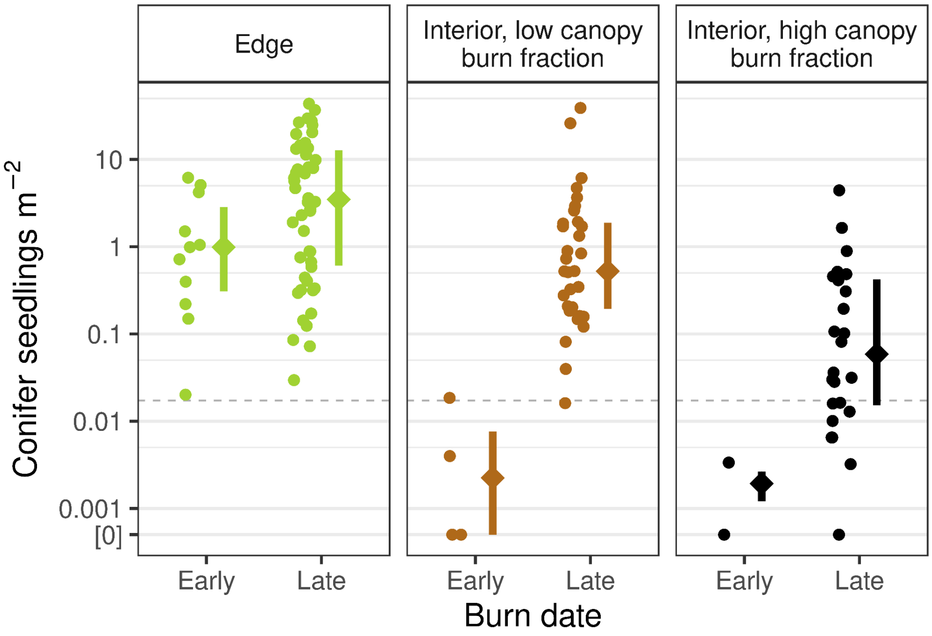

Fig. S7. Equivalent of Fig. 2A, using only interior plots > 100 m from surviving reproductive trees.

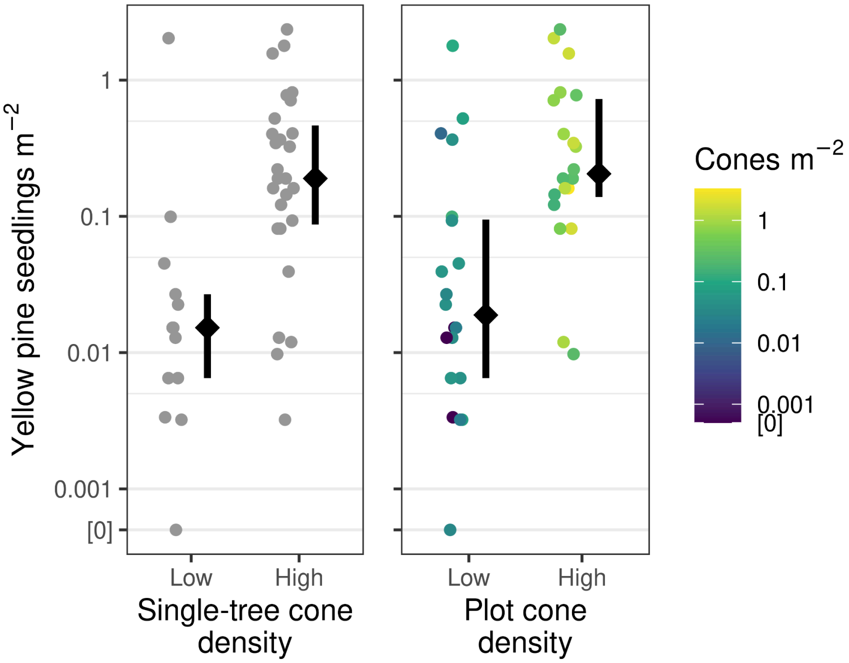

Fig. S8. Equivalent of Fig. 2B, using only interior plots > 100 m from surviving trees.

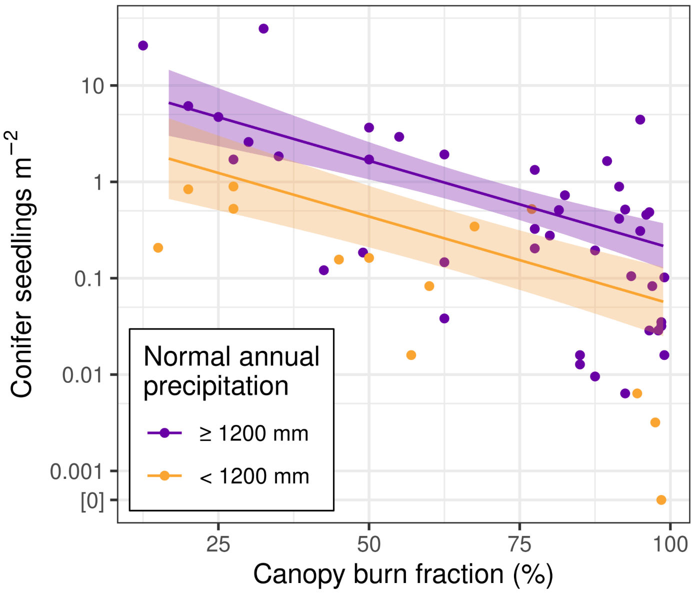

Fig. S9. Equivalent of Fig. 3A, using only interior plots > 100 m from surviving trees.

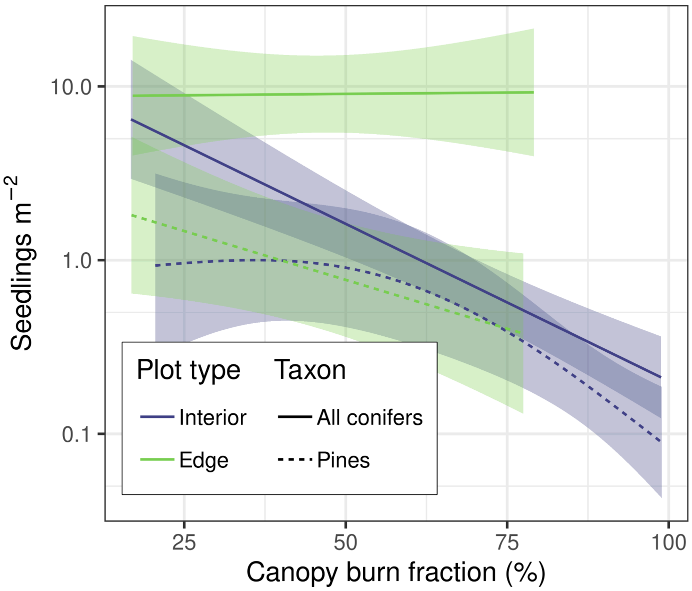

Fig. S10. Equivalent of Fig. 3B, using only interior plots > 100 m from surviving trees.

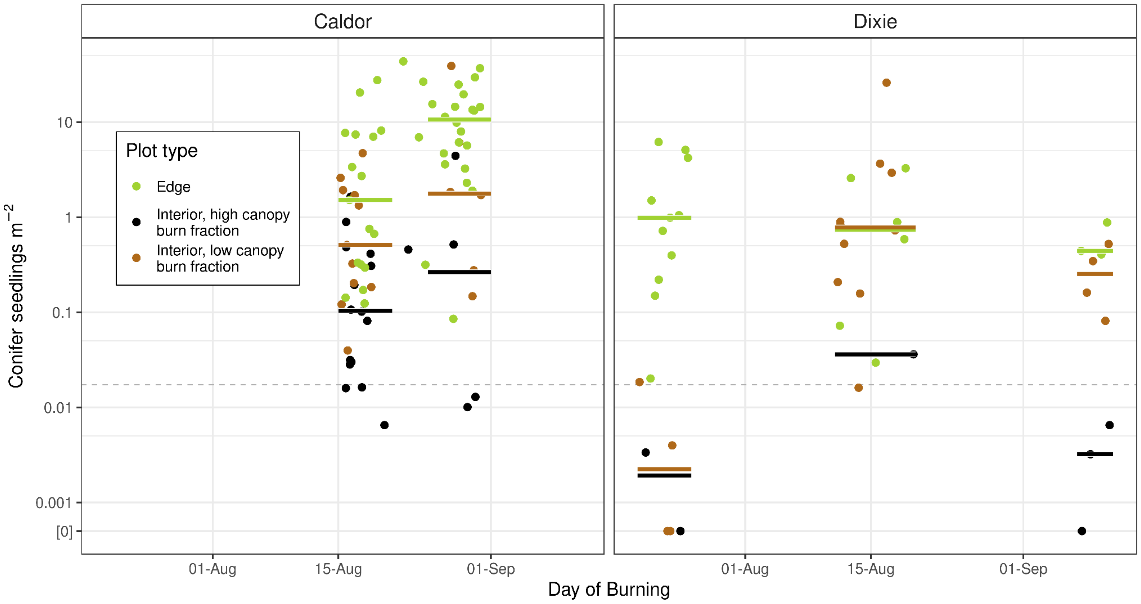

Fig. S11. Equivalent of Fig. S6, using only plots > 100 m from surviving trees.

Section S3: Supplemental tables

Table S1. Observations of conifer reproduction summarized by plot type.

Plot location	Canopy burn fraction	Burn date	Plot count	Seedling density (seedlings ha^{-1}) (median and interquartile range)				Median cone density (cones ha^{-1}) (median and interquartile range)		Nearest canopy dominant tree is highly reproductive (% of plots)	
				PIPJ	ABIES	CADE	ALL	PIPJ	PSME	PIPJ	PSME
Interior	Low	Early	8	16 (0, 34)	0 (0, 42)	0 (0, 0)	38 (24, 84)	2189 (386, 2961)	1567 (0, 5611)	75	12
		Late	43	1608 (872, 3921)	553 (97, 12426)	0 (0, 0)	5227 (1524, 17746)	1543 (323, 3259)	0 (0, 0)	74	7
	High	Early	9	0 (0, 34)	0 (0, 0)	0 (0, 0)	0 (0, 34)	896 (348, 2637)	547 (0, 746)	44	11
		Late	36	215 (65, 714)	65 (0, 591)	0 (0, 0)	439 (126, 3861)	448 (100, 2152)	0 (0, 0)	39	6
Edge	Low	–	55	1715 (542, 8276)	25591 (808, 60866)	652 (33, 2870)	33716 (6273, 90106)	498 (25, 2264)	0 (0, 0)	82	16
	High	–	6	450 (191, 2622)	3075 (669, 16132)	17 (0, 34)	8000 (755, 21023)	597 (87, 2376)	0 (0, 0)	83	0

Notes: "Early" burned plots burned before August 1, while "Late" burned plots burned after. "Low" canopy burn fraction plots had less than 85% canopy burn fraction, while "High" canopy burn fraction plots had greater. Densities are summarized using the median due to the highly right-skewed distribution. The median density and interquartile range of sugar pine and Douglas-fir seedlings across all plot categories was 0 (i.e., > 50% of plots had zero seedlings of the species). Similarly, the median cone density and interquartile range of sugar pine was 0. Cones of firs and incense cedar either disintegrate in the canopy as they open (firs) or are too small to reliably find on the ground (incense cedar) and were not surveyed. Classification of the nearest canopy-dominant tree as highly reproductive or not was performed as described in "Field data collection", *Methods* section. PIPJ: Ponderosa and Jeffrey pines; ABIES: firs (*Abies* sp.); CADE: incense cedar; PILA: sugar pine; PSME: Douglas-fir; ALL: all conifers.

Table S2. Theoretical densities of deposited seeds along a transect from a green forest edge, assuming seeds originate only from the green trees (i.e., no canopy seed survival in fire-killed trees), alongside observed seedling densities in low canopy burn fraction plots burned within different date ranges. Theoretical seed densities are calculated following ref. (Greene and Johnson 1996) (see Materials and Methods) and are presented as a percentage of the expected density at 30 m. Observed seedling density is presented as a percentage of the density in the edge plots burned in the same date range (with both interior and edge plot densities summarized using the median prior to computing percentages). Median distance from edge for edge plots was 43.3 m for plots burning before 1 August, 29.9 m for plots burning after 1 August, and 31.2 m for plots burning between 10 and 20 August. All interior plots were > 60 m from the nearest surviving tree. In most cases, the nearest surviving tree was farther than this distance, and the nearest green edge was much farther still.

**Distance from edge (m)**	**Theoretical relative seed density (%)**			**Plot type**	**Observed relative seedling density (%)**		
	White fir	Ponderosa pine	Jeffrey pine		< 1 August (n = 11 edge, 8 interior)	> 1 August (n = 50 edge, 40 interior)	10-20 August (n = 21 edge, 25 interior)
30	100	100	100	Edge	100	100	100
100	22.2	20.8	15.4				
200	3.4	3.1	1.6	Interior	0.38	15.0	48
300	0.7	0.5	0.2				
400	0.1	0.1	0.0				

Table S3. Equivalent of Table 1, using only interior plots > 100 m from surviving reproductive trees.

Plot type	Species group	Sample size (plots)	Full model effective degrees of freedom	Deviance explained (%)		Deviance explained by canopy burn fraction (%)
				Full model	Full model minus "canopy burn fraction"	
Interior	All conifers	55	3.00	47.9	12.6	35.3
	Pines	45	5.55	25.7	3.4	22.3
Edge	All conifers	28	3.43	23.3	23.2	0.1
	Pines	23	5.26	40.5	30.7	9.8

Table S4. Equivalent of Table S2, using only interior plots > 100 m from surviving trees.

Distance from edge (m)	Theoretical relative seed density (%)			Plot type	Observed relative seedling density (%)		
	White fir	Ponderosa pine	Jeffrey pine		< 1 August (n = 11 edge, 4 interior)	> 1 August (n = 50 edge, 31 interior)	10-20 August (n = 21 edge, 21 interior)
30	100	100	100	Edge	100	100	100
100	22.2	20.8	15.4	Interior	0.20	15.1	48
200	3.4	3.1	1.6				
300	0.7	0.5	0.2				
400	0.1	0.1	0.0				

Table S5. Plot summary metrics based on the full dataset including interior plots > 60 m from surviving reproductive trees (reported in main text) and, for comparison, only interior plots > 100 m from surviving reproductive trees. In general, excluding interior plots < 100 m from surviving trees strengthened the signals suggestive of canopy seed survival (indicated by bold text) or had no substantial effect.

	**Minimum distance to nearest surviving tree (> 5% prefire foliage remaining green)**	
**Metric name**	**60m**	**100m**
Median conifer seedling density (seedlings m^{-2}) across all interior plots	0.104	**0.203**
Median conifer seedling density (seedlings m^{-2}) across interior plots burning prior to August	0.0032	**0.0017**
Fold change in yellow pine seedling density between interior plots where conspecific cones were abundant vs. where they were scarce	8	**10.8**
Fold change in model-fitted conifer seedling density (seedlings m^{-2}) from 25% to 95% canopy burn fraction in later-burned interior plots	16	**18**
Fold change in model-fitted pine seedling density (seedlings m^{-2}) from 25% to 95% canopy burn fraction in later-burned interior plots	7.5	**8**
White fir proportion (%) of seedlings in later-burned interior plots at low (0-50%) canopy burn fraction	76	77
Yellow pine proportion (%) of seedlings in later-burned interior plots at low (0-50%) canopy burn fraction	24	23
White fir proportion (%) of seedlings in later-burned interior plots at high (80-100%) canopy burn fraction	27	27
Yellow pine proportion (%) of seedlings in later-burned interior plots at high (80-100%) canopy burn fraction	73	73
Yellow pine proportion (%) of seedlings in later-burned interior plots	81	80
White fir proportion (%) of seedlings in later-burned interior plots	19	20
Pre-fire overstory yellow pine proportion (%) across all later-burning interior plots	42	43
Pre-fire overstory white fir proportion (%) across all later-burning interior plots	36	37
Pre-fire overstory incense cedar proportion (%) across all later-burning interior plots	12	11
Pre-fire overstory sugar pine proportion (%) across all later-burning interior plots	7	7
Pre-fire overstory Douglas-fir proportion (%) across all later-burning interior plots	2	2
Seedling density (seedlings m^{-2}) exceeded in at least one plot by white fir, Douglas-fir, sugar pine, and yellow pines	0.1	0.1
Number of low canopy burn fraction interior plots	45	37
Number of high canopy burn fraction interior plots	51	24

Section S4: Appendix S1 references

Coop, J. D., S. A. Parks, C. S. Stevens-Rumann, S. M. Ritter, and C. M. Hoffman. 2022. Extreme fire spread events and area burned under recent and future climate in the western USA. Global Ecology and Biogeography 31:1949–1959.

Davies, D., G. Ederer, O. Olsina, M. Wong, M. Cechini, and R. Boller. 2019. NASA's Fire Information for Resource Management System (FIRMS): Near Real-Time Global Fire Monitoring Using Data from MODIS and VIIRS. Page EARSel Forest Fires SIG Workshop.

Eidenshink, J. C., B. Schwind, K. Brewer, Z.-L. Zhu, B. Quayle, and S. M. Howard. 2007. A project for monitoring trends in burn severity. Fire ecology 3:3–21.

Greene, D. F., and E. A. Johnson. 1996. Wind Dispersal of Seeds from a Forest Into a Clearing. Ecology 77:595–609.

North, M. P., J. T. Stevens, D. F. Greene, M. Coppoletta, E. E. Knapp, A. M. Latimer, C. M. Restaino, R. E. Tompkins, K. R. Welch, R. A. York, D. J. N. Young, J. N. Axelson, T. N. Buckley, B. L. Estes, R. N. Hager, J. W. Long, M. D. Meyer, S. M. Ostoja, H. D. Safford, K. L. Shive, C. L. Tubbesing, H. Vice, D. Walsh, C. M. Werner, and P. Wyrsch. 2019. Tamm Review: Reforestation for resilience in dry western U.S. forests. Forest Ecology and Management 432:209–224.

Parks, S. A. 2014. Mapping day-of-burning with coarse-resolution satellite fire-detection data. International Journal of Wildland Fire 23:215–223.

Welch, K. R., H. D. Safford, and T. P. Young. 2016. Predicting conifer establishment post wildfire in mixed conifer forests of the North American Mediterranean-climate zone. Ecosphere 7:e01609.
